# Respiratory physiology of COVID-19-induced respiratory failure compared to ARDS of other etiologies

**DOI:** 10.1186/s13054-020-03253-2

**Published:** 2020-08-28

**Authors:** Domenico Luca Grieco, Filippo Bongiovanni, Lu Chen, Luca S. Menga, Salvatore Lucio Cutuli, Gabriele Pintaudi, Simone Carelli, Teresa Michi, Flava Torrini, Gianmarco Lombardi, Gian Marco Anzellotti, Gennaro De Pascale, Andrea Urbani, Maria Grazia Bocci, Eloisa S. Tanzarella, Giuseppe Bello, Antonio M. Dell’Anna, Salvatore M. Maggiore, Laurent Brochard, Massimo Antonelli

**Affiliations:** 1 Department of Emergency, Intensive Care Medicine and Anesthesia, Fondazione Policlinico Universitario A. Gemelli IRCCS, Rome, Italy; 2 Istituto di Anestesiologia e Rianimazione, Università Cattolica del Sacro Cuore, Rome, Italy; 3 Keenan Centre for Biomedical Research, Li Ka Shing Knowledge Institute, St. Michael’s Hospital, Toronto, Canada; 4 Interdepartmental Division of Critical Care Medicine, University of Toronto, Toronto, Canada; 5 Department of Basic Biotechnological Science, Università Cattolica del Sacro Cuore, Rome, Italy; 6 Department of Laboratory and Infectious Diseases, Fondazione Policlinico Universitario A. Gemelli IRCCS, Rome, Italy; 7 Department of Medical, Oral and Biotechnological Sciences, School of Medicine and Health Sciences, Section of Anesthesia, Analgesia, Perioperative and Intensive Care, SS. Annunziata Hospital, Gabriele d’Annunzio University of Chieti-Pescara, Chieti, Italy

**Keywords:** COVID-19, ARDS, Respiratory mechanics, PEEP, Alveolar recruitment

## Abstract

**Background:**

Whether respiratory physiology of COVID-19-induced respiratory failure is different from acute respiratory distress syndrome (ARDS) of other etiologies is unclear. We conducted a single-center study to describe respiratory mechanics and response to positive end-expiratory pressure (PEEP) in COVID-19 ARDS and to compare COVID-19 patients to matched-control subjects with ARDS from other causes.

**Methods:**

Thirty consecutive COVID-19 patients admitted to an intensive care unit in Rome, Italy, and fulfilling moderate-to-severe ARDS criteria were enrolled within 24 h from endotracheal intubation. Gas exchange, respiratory mechanics, and ventilatory ratio were measured at PEEP of 15 and 5 cmH_2_O. A single-breath derecruitment maneuver was performed to assess recruitability. After 1:1 matching based on PaO_2_/FiO_2_, FiO_2_, PEEP, and tidal volume, COVID-19 patients were compared to subjects affected by ARDS of other etiologies who underwent the same procedures in a previous study.

**Results:**

Thirty COVID-19 patients were successfully matched with 30 ARDS from other etiologies. At low PEEP, median [25th–75th percentiles] PaO_2_/FiO_2_ in the two groups was 119 mmHg [101–142] and 116 mmHg [87–154]. Average compliance (41 ml/cmH_2_O [32–52] vs. 36 ml/cmH_2_O [27–42], *p* = 0.045) and ventilatory ratio (2.1 [1.7–2.3] vs. 1.6 [1.4–2.1], *p* = 0.032) were slightly higher in COVID-19 patients. Inter-individual variability (ratio of standard deviation to mean) of compliance was 36% in COVID-19 patients and 31% in other ARDS. In COVID-19 patients, PaO_2_/FiO_2_ was linearly correlated with respiratory system compliance (*r* = 0.52 *p* = 0.003). High PEEP improved PaO_2_/FiO_2_ in both cohorts, but more remarkably in COVID-19 patients (*p* = 0.005). Recruitability was not different between cohorts (*p* = 0.39) and was highly inter-individually variable (72% in COVID-19 patients and 64% in ARDS from other causes). In COVID-19 patients, recruitability was independent from oxygenation and respiratory mechanics changes due to PEEP.

**Conclusions:**

Early after establishment of mechanical ventilation, COVID-19 patients follow ARDS physiology, with compliance reduction related to the degree of hypoxemia, and inter-individually variable respiratory mechanics and recruitability. Physiological differences between ARDS from COVID-19 and other causes appear small.

## Background

Around 5% of patients affected by the novel 2019 coronavirus disease (COVID-19) require intensive care unit (ICU) admission due to acute respiratory distress syndrome (ARDS), with a case-fatality rate ranging between 30 and 60% [1–8]. Invasive mechanical ventilation is required in most of the patients to treat gas exchange abnormalities and represents the mainstay of supportive therapy [4, 7, 9]. In this setting, mechanical ventilation is aimed at restoring adequate gas exchange while limiting ventilator-induced lung injury (VILI) [10]. During ARDS, proper ventilatory management reduces the risk of VILI and is among the potentially modifiable factors capable of improving survival [11].

The effects of ventilator settings (tidal volume, positive end-expiratory pressure [PEEP]) on VILI and clinical outcome are mediated by respiratory mechanics that have wide inter-individual variability [12–14]. Thorough understanding of respiratory mechanics is essential to limit the risk of VILI and, possibly, improve clinical outcome [15, 16]. Some reports suggested that patients with COVID-19 ARDS may have different phenotypes (high vs. low elastance), independently from gas exchange [17]. This could have important implications regarding ventilator management. Some authors claim that COVID-19 patients (or, at least, part of them) may not necessarily benefit from usual ARDS management [18]. However, whether or not the heterogeneity in respiratory mechanics observed in COVID-19 patients is different from conventional ARDS remains unclear [19–23].

We assessed respiratory mechanics, potential for lung recruitment, and PEEP effects in 30 consecutive mechanically ventilated patients with COVID-19-induced moderate-to-severe ARDS. After 1:1 matching based on the degree of oxygenation impairment at same PEEP and FiO_2_, we then compared these results with those obtained from a multicenter cohort of patients with ARDS of other origins who underwent the same procedures in a previous study.

## Methods

This prospective study was conducted in the dedicated COVID-19 ICU of a tertiary care university hospital in Italy during March 2020. Approval was obtained by local institutional review board, and informed consent was obtained according to committee recommendations.

### Patients

We screened all consecutive adult patients admitted to ICU between March 16 and 27, 2020, who were intubated due to acute hypoxemic respiratory failure with confirmed molecular diagnosis of COVID-19 (positive real-time polymerase chain reaction for viral RNA performed on an upper or lower respiratory tract specimen). Patients fulfilling criteria for moderate and severe ARDS according to the Berlin definition (i.e., PaO_2_/FiO_2_ ratio ≤ 200, measured at PEEP = 5 cmH_2_O) [24, 25] were enrolled within 24 h from endotracheal intubation.

Exclusion criteria were as follows: (1) age < 18 years, (2) undrained pneumothorax, and (3) hemodynamic instability, defined as > 30% increase in vasopressor requirement during the previous 6 h or norepinephrine > 0.5 μg/kg/min.

### Procedures and measurements

For each patient, demographics, comorbidities, and 28-day clinical outcome were recorded.

All measurements were conducted in the supine semi-recumbent position within 24 h from endotracheal intubation, before any session of prone positioning. All patients were sedated and paralyzed with cisatracurium continuous infusion at a standard dose of 35 mg/h [26]. Mechanical ventilation was applied in the volume-controlled mode with a heat and moisture exchanger, with the following settings: tidal volume 6 ml/kg of predicted body weight (PBW), inspiratory flow 60 l/min, inspiratory pause 0.3 s, respiratory rate titrated to obtain pH > 7.30 and < 35 breaths per minute, and FiO_2_ titrated to achieve SpO_2_ between 90 and 96%.

Two PEEP levels were tested in a sequential order: 15 (or the highest PEEP to obtain plateau pressure ≤ 28 cmH_2_O) and 5 cmH_2_O. After 30 min of ventilation with PEEP = 15 cmH_2_O, arterial blood gasses and hemodynamics were recorded. Inspiratory (1.5 s) and expiratory (4 s) holds were performed, and the following parameters collected:

- *Respiratory mechanics*: Peak airway pressure, plateau pressure, and total PEEP were measured. Driving pressure, respiratory system compliance, and its PBW-indexed value were computed. Ventilatory ratio, which is an estimate of dead space fraction, was calculated (tidal volume × respiratory rate × PaCO_2_)/(PBW × 100 × 37.5) [27].
- *Recruitability*: A single-breath derecruitment maneuver was performed by decreasing PEEP by 10 cmH_2_O [28, 29]; exhaled tidal volume after PEEP lowering was recorded, and recruitment-to-inflation ratio was computed [29]—patients with recruitment-to-inflation ratio ≥ 0.5 were considered having high recruitability.

Afterwards, ventilation was resumed with previous settings and PEEP = 5 cmH2O. After 30 min, blood gasses, hemodynamics, and respiratory mechanics were re-assessed as described. Lastly, low-flow (5 l/min) inflation was performed after prolonged exhalation to assess airway closure, and airway opening pressure was recorded if present [30–33].

### Comparison with non-COVID-19 ARDS

COVID-19 patients were compared to a cohort of subjects with moderate-to-severe ARDS from other etiologies who underwent exactly the same procedures in a previous study [29]. Two investigators (DLG and LC) were directly involved in patients’ enrolment in both studies. This ensures reproducibility of the measurements and consistency in ventilator settings and circuit setup. COVID-19 patients were matched in 1:1 ratio to patients from the non-COVID-19 ARDS cohort. Matching was based on PaO_2_/FiO_2_ (± 20 mmHg), FiO_2_ (± 0.2), PEEP (± 3 cmH_2_O), and tidal volume (± 1.5 ml/kg of predicted body weight). For matching, priority was given to PaO_2_/FiO_2_ (100% adherence to the criterion), followed by FiO_2_ (93% adherence to the criterion, for “2 matches” criterion increased to ± 0.4), tidal volume (93% adherence to the criterion, for “2 matches” criterion increased to ± 2.5 ml/kg), and PEEP (90% adherence to the criterion, for “3 matches” criterion increased to ± 5 cmH_2_O). Individual data of matched subjects are provided in supplementary table 1.

### Endpoints

The aims of the study were to describe respiratory mechanics, potential for lung recruitment, and response to PEEP in COVID-19-induced ARDS patients and to compare these features to those of patients affected by ARDS of other causes.

### Sample size and statistical analysis

At the time of study design, systematic data on respiratory mechanics in COVID-19 patients were lacking. Hence, a convenience sample of 30 consecutive patients was chosen to provide a timely report. Categorical data are reported as number of events (%), and continuous data are displayed as medians [interquartile range]. Comparisons of continuous variables at the two PEEP levels were performed with the *T* test for paired samples: mean differences [95% confidence intervals, CI95%] are displayed for most significant results. Categorical variables were compared with the McNemar test. Inter-individual variability was calculated as the ratio of standard deviation to mean of the measurements.

Comparisons of continuous variables between COVID-19 and ARDS cohort were performed with the *T* test for independent samples: mean differences are displayed for significant results. Categorical variables were compared with the chi-square or Fisher exact test, as appropriate.

Correlations were assessed with Pearson’s correlation: *r* and *p* are provided for each comparison. Results with two-tailed *p* ≤ 0.05 were considered statistically significant. Statistical analysis was performed with SPSS 20.0 (IBM Corporation, Armonk, NY, USA). Manuscript figures were prepared with GraphPad Prism (La Jolla, CA, USA).

## Results

### COVID-19 cohort

Thirty patients were enrolled. Demographics and relevant clinical characteristics are reported in Table 1. Twenty-three (77%) patients met the criteria for moderate ARDS, and 7 (23%) for severe ARDS.

**Table 1:** Demographics and baseline characteristics of enrolled patients

	COVID-19 cohort, *n* = 30	Non-COVID-19 cohort, *n* = 30
Age, years	70 [63–77]	61 [51–69]
Female sex, no. (%)	7 (23)	4 (13)
Height, cm	170 [170–175]	171 [167–180]
Predicted body weight, kg	66 [62–75]	66 [59–75]
Body mass index, kg/m^2^	28 [25–29]	33 [27–40]
SOFA at study inclusion	8 [7–10]	14 [10–15]
SAPS II	45 [34–58]	
Comorbidities, no. (%)		
Hypertension	19 (63)	
Active cancer	3 (10)	
Chronic obstructive pulmonary disease	3 (10)	
Diabetes mellitus	2 (7)	
Coronary artery disease	1 (3)	
Other	10 (33)	
ARDS risk factors, no. (%)		
Pneumonia	30 (100)	10 (33)
Aspiration	0 (0)	4 (13)
Extrapulmonary sepsis	0 (0)	4 (13)
Trauma	0 (0)	2 (7)
Other	0 (0)	10 (33)
Noninvasive respiratory support before intubation, no. (%)	20 (67)	
Duration of noninvasive respiratory support before intubation, hours	19 [9–63]	
ARDS severity at enrollment, no. (%)		
Moderate (PaO_2_/FiO_2_ ratio 101–200 mmHg)	23 (77)	22 (73)
Severe (PaO_2_/FiO_2_ ratio ≤ 100 mmHg)	7 (23)	8 (27)
Prone positioning during the ICU stay, no. (%)	21 (70)	
Acute kidney failure, no. (%)	15 (50)	
ICU-acquired infection, no. (%)	9 (30)	
Pneumothorax, no. (%)	4 (13)	
Tracheostomy, no. (%)	8 (27)	
28-day outcome, no. (%)		
Dead	19 (63)	9 (30)
Alive, receiving mechanical ventilation	3 (10)	
Alive, breathing unassisted	8 (27)	

Data expressed in median [interquartile range], if not otherwise specified

### Matched cohorts

Thirty patients from the historical ARDS cohort were successfully matched to COVID-19 patients (individual data provided in supplementary Table 1, demographics in Table 1). At low PEEP, median PaO_2_/FiO_2_ was 119 mmHg [101–142] in COVID-19 patients and 116 mmHg [87–154] in patients with ARDS from other etiologies. FiO_2_ was not different between cohorts (*p* = 0.51), while tidal volume was slightly higher, and PEEP lower, in patients with COVID-19 than controls. Although statistically significant, mean differences between cohorts were clinically negligible: 0.3 ml/kg [CI95% 0–0.6] and 0.9 cmH_2_O [CI95% 0.1–1.7], respectively. All COVID-19 patients were studied within 24 h from endotracheal intubation. Patients in the historical cohort underwent study procedures after a median time from ICU admission of 5 [3–10] days.

### Respiratory mechanics

**Fig. 1:**
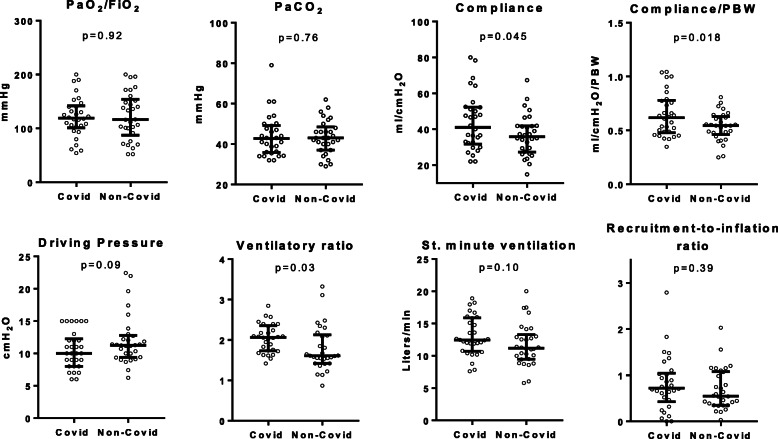
Respiratory mechanics. Individual values, medians, and interquartile range showing the distribution of respiratory variables in the matched cohorts. For each variable, COVID-19 patients’ values are compared to those for matched non-COVID-19 ARDS patients, as detailed in text. PaO_2_/FiO_2_ ratio, PaCO_2_, respiratory system compliance, driving pressure, ventilatory ratio, and standardized minute ventilation were measured at low PEEP. Ventilatory ratio, compliance, and its predicted body weight-indexed value were slightly higher in COVID-19 patients than in ARDS of other etiologies. PBW, predicted body weight

These results are displayed in Fig. 1 and Table 2.

**Table 2:** Respiratory mechanics

	Low PEEP			High PEEP		
	COVID-19	Non-COVID-19	*p*	COVID-19	Non-COVID-19	*p*
Set PEEP, cmH_2_O	5 [5–5]*	5 [5–8]^§^	0.031	15 [15–15]*	15 [15–18]^§^	0.011
Total PEEP, cmH_2_O	5 [5–6]*	8 [6–9]^§^	< 0.001	15 [15–16]*	16 [15–18]^§^	0.001
Tidal volume, ml	431 [395–473]	414 [370–443]	0.15	431 [395–473]	417 [357–445]	0.20
Tidal volume/PBW, ml/kg	6.4 [6–6.8]	6 [5.7–6.3]	0.037	6.4 [6–6.8]	6 [5.8–6.3]	0.036
Respiratory rate, breaths/minute	28 [26–30]	26 [24–30]	0.16	28 [26–30]	26 [24–30]	0.11
PaO_2_/FiO_2_, mmHg	119 [101–142]*	116 [87–154]^§^	0.92	165 [132–196]*	150 [121–192]^§^	0.049
pH	7.35 [7.29–7.42]	7.37 [7.33–7.40]	0.63	7.35 [7.32–7.42]	7.36 [7.32–7.40]	0.45
PaCO_2_, mmHg	43 [37–49]	43 [37–49]	0.76	43 [36–49]	45 [35–49]	0.91
Ventilatory ratio	2.1 [1.7–2.3]	1.6 [1.4–2.1]	0.032	2.1 [1.7–2.4]	1.7 [1.4–2.2]	0.08
Standardized minute ventilation, liters/minute	12.4 [10.7–15.6]	11.1 [9.5–13.3]	0.12	12.4 [10.7–15.9]	11.5 [9.7–14.2]	0.29
Peak pressure, cmH_2_O	29 [23–32]*	33 [29–39]^§^	0.003	39 [36–41]*	41 [39–46]^§^	0.043
Plateau pressure, cmH_2_O	15 [14–17]*	19 [16–22]^§^	< 0.001	26 [25–29]*	30 [28–33]^§^	< 0.001
Inspiratory resistance, cmH_2_O/liters/second	12 [10–14]	14 [11–16]^§^	0.09	12 [10–14]	12 [10–14]^§^	0.55
Driving pressure, cmH_2_O	10 [8–12]	11 [9–13]^§^	0.09	10 [9–14]	13 [11–17]^§^	0.007
Patients with driving pressure ≤ 14 cmH_2_O, no. (%)	24 (80)	25 (83)	1	24 (80)	21 (70)	0.55
Respiratory system compliance, ml/cmH_2_O	41 [32–52]	36 [27–42]^§^	0.045	39 [27–53]	32 [23–40]^§^	0.003
Respiratory system compliance/PBW, ml/cmH_2_O/kg	0.62 [0.48–0.78]	0.54 [0.46–0.63]^§^	0.018	0.57 [0.45–0.75]	0.47 [0.37–0.56]^§^	< 0.001
Arterial pressure, mmHg						
Systolic	130 [112–140]*	124 [110–131]^§^	0.27	118 [110–126]*	116 [104–126]^§^	0.71
Diastolic	70 [57–80]	60 [55–64]	0.017	65 [54–70]	57 [54–62]	0.08
Heart rate, beats per minute	78 [70–93]	82 [74–101]	0.15	80 [70–92]	85 [75–103]	0.15

Data are expressed as medians [interquartile range], if not otherwise specified

*p < 0.05 for the comparison between low and high PEEP within the COVID-19 cohort

^§^*p* < 0.05 for the comparison between low and high PEEP within the non-COVID-19 cohort

**Fig. 2:**
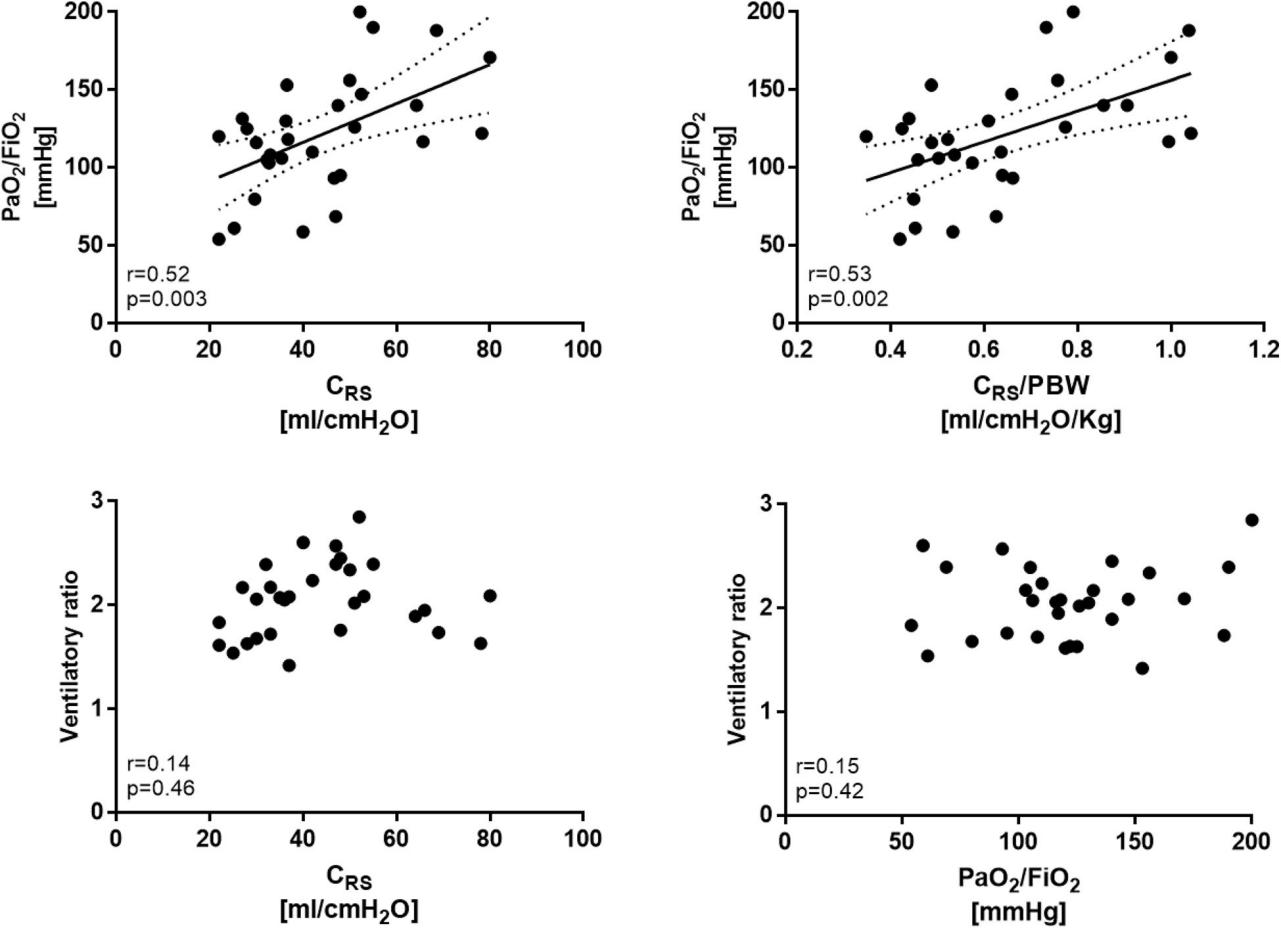
Relationships between PaO_2_/FiO_2_, respiratory system compliance, and ventilatory ratio in COVID-19 ARDS patients. In the COVID-19 cohort, respiratory system compliance and its PBW-indexed value were linearly related to PaO_2_/FiO_2_ ratio (*upper panels*). Ventilatory ratio was not related to PaO_2_/FiO_2_ ratio nor to respiratory system compliance (lower panels). C_RS_, respiratory system compliance; PBW, predicted body weight

Airway closure in COVID-19 cohort was less frequent than in ARDS from other etiologies: 2 (7%) vs. 10 (30%) patients (*p* = 0.021). At low PEEP, inter-individual variability of respiratory system compliance was 36% in COVID-19 patients and 31% in ARDS from other causes. Average respiratory system compliance and respiratory system compliance/PBW were slightly higher in patients with COVID-19 than in those affected by other ARDS: mean differences were 7 ml/cmH_2_O [CI95% 0–14] and 0.11 ml/cmH_2_O/kg [CI95% 0.2–0.20], respectively. This was not associated to statistically significant differences in the driving pressure (*p* = 0.098). In both cohorts, twenty-four (80%) patients showed driving pressure equal or lower than 14 cmH_2_O. In the COVID-19 cohort, respiratory system compliance (*r* = 0.52, *p* = 0.003) and respiratory system compliance/PBW (*r* = 0.53, *p* = 0.002) were linearly related to PaO_2_/FiO_2_ (Fig. 2).

In COVID-19 cohort, ventilatory ratio was higher than in ARDS from other etiologies (mean difference 0.3 [CI95% 0–0.6], *p* = 0.032). In COVID-19 cohort, ventilatory ratio was not related to PaO_2_/FiO_2_ (*p* = 0.42) nor to respiratory system compliance (*p* = 0.46).

### Response to PEEP—gas exchange and respiratory mechanics

**Fig. 3:**
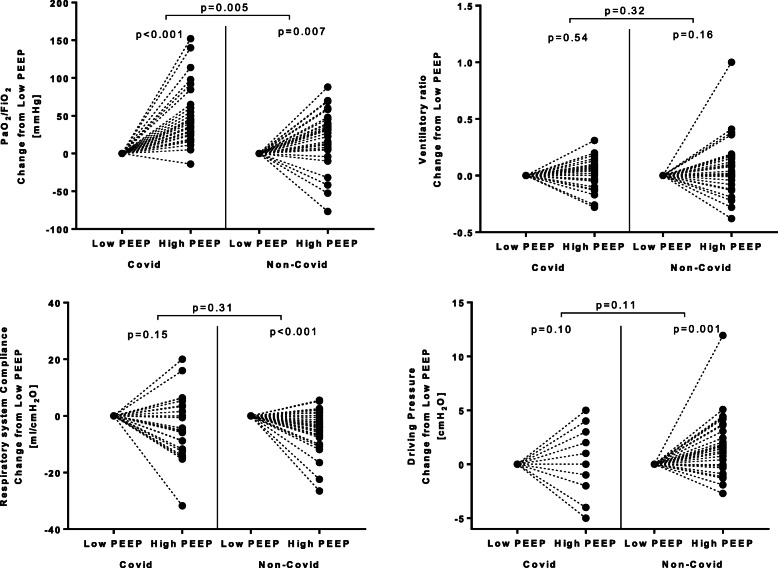
Response to PEEP. Before-and-after plots showing the effects of high PEEP on PaO_2_/FiO_2_ ratio (*upper left* panel), ventilatory ratio (*upper right* panel), respiratory system compliance (*lower left* panel), and driving pressure (*lower right* panel). In both groups, PaO_2_/FiO_2_ ratio increased at increasing PEEP, but the increase was significantly higher in the COVID-19 cohort (see also Table 2). Ventilatory ratio could either increase, decrease, or remain unchanged, with no significant difference between cohorts. At high PEEP, compliance increased and driving pressure decreased in non-COVID-19 patients, while no changes were detected in COVID-19 patients. *Black dots* represent individual patients before and after the increase in PEEP, and individual changes are traced by *dotted lines*. C_RS_, respiratory system compliance

**Fig. 4:**
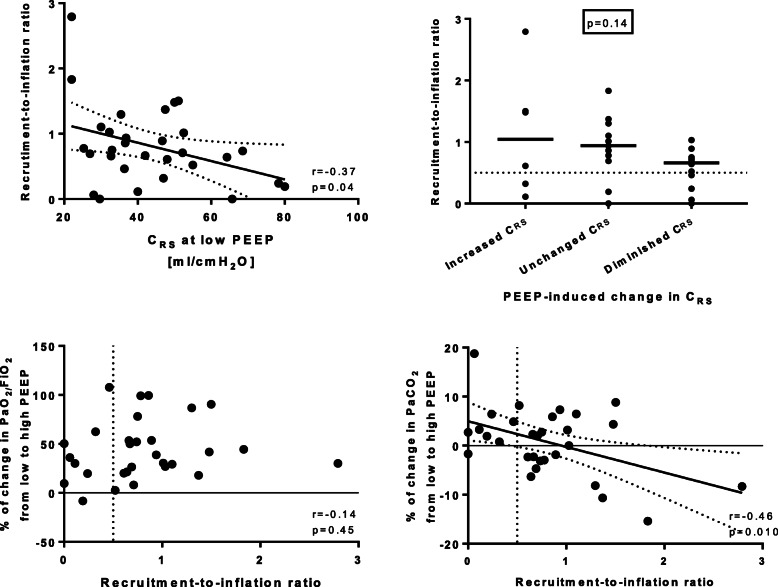
Potential for lung recruitment in COVID-19 ARDS patients. In COVID-19 patients, recruitment-to-inflation ratio was inversely related to respiratory system compliance at low PEEP (*upper left* panel, Pearson’s correlation and linear regression), meaning that patients with lower baseline compliance displayed the highest potential for lung recruitment. Importantly, lung recruitability was not related to changes in respiratory system compliance (and driving pressure) induced by PEEP. With PEEP, compliance could either increase, decrease, or remain unchanged (change in compliance was defined clinically relevant when >5 ml/cmH2O), independently from the recruitment-to-inflation ratio, as shown in the *upper right* panel. The changes in PaO_2_/FiO_2_ induced by PEEP were independent from recruitability (*lower left* panel), while PEEP-induced PaCO_2_ changes were weakly but significantly related to the recruitment-to-inflation ratio (*lower right* panel,). C_RS_, respiratory system compliance

These results are displayed in Table 2 and Figs. 3 and 4.

High PEEP yielded improvement in PaO_2_/FiO_2_ in both cohorts. The PEEP-induced improvement in PaO_2_/FiO2 was greater in COVID-19 patients than among subjects with non-COVID-19 ARDS: median PEEP-induced changes in PaO_2_/FiO_2_ were 42 mmHg [24–62] vs. 27 mmHg [5–42], respectively (*p* = 0.005). In 27 (97%) patients of COVID-19 cohort, high PEEP yielded improvement in PaO_2_/FiO_2_.

In both cohorts, PEEP did not affect PaCO_2_ nor ventilatory ratio. In COVID-19 patients, respiratory system compliance and driving pressure did not change with high PEEP: in ARDS from non-COVID-19 etiology, high PEEP reduced compliance by 5 ml/cmH_2_O [CI95% 2–8] and increased driving pressure by 2 cmH_2_O [CI95% 1–3].

In both cohorts, high PEEP caused reduction in systolic arterial pressure, without affecting heart rate and diastolic pressure.

### Response to PEEP—recruitability

Median recruitment-to-inflation ratio (i.e., recruitability) was 0.73 [0.43–1.04] in COVID-19 patients and 0.55 [0.35–1.08] in ARDS from other causes (*p* = 0.39). Inter-individual variability of the recruitment-to-inflation ratio was 72% and 64%, respectively. Recruitment-to-inflation ratio was greater than 0.5 in 22 COVID-19 patients (73%) vs. 17 patients (57%) from the non-COVID-19 ARDS cohort (*p* = 0.28).

In COVID-19 patients, recruitment-to-inflation ratio had a weak but significant inverse correlation with respiratory system compliance recorded at low PEEP (*r* = − 0.37, *p* = 0.04). Recruitment-to-inflation ratio had no relationship with the change in PaO_2_/FiO_2_ caused by high PEEP (*p* = 0.45) but had a reverse linear relationship with the change in PaCO_2_ due to PEEP (*r* = − 0.46 *p* = 0.010) (Fig. 4).

Recruitment-to-inflation was not different between patients who showed increased, decreased, or unchanged respiratory system compliance (and driving pressure) with high PEEP (*p* = 0.14).

### Clinical outcome

At 28 days, 19 (63%) patients in the COVID-19 cohort had died and 3 (10%) were still on mechanical ventilation.

## Discussion

The result of this matched-cohort study can be summarized as follows:

- In COVID-19 patients, the severity of hypoxemia was related to respiratory system compliance reduction. This suggests that aeration loss is a relevant mechanism of hypoxemia.
- Similarly to ARDS from other causes, respiratory mechanics of COVID-19 patients was highly heterogeneous.
- Average respiratory system compliance and ventilatory ratio of COVID-19 patients were slightly higher than those of ARDS from other etiologies. Although statistically significant, differences appear clinically small.
- The potential for PEEP-induced lung recruitment was variable. Average recruitability was similar to ARDS from non-COVID etiology. COVID-19 patients showed frank oxygenation response to PEEP, independently from recruitability.

### Respiratory mechanics

Few data are available about respiratory mechanics and response to PEEP COVID-19 patients with acute respiratory failure [18–20, 34]. Our study compared respiratory mechanics and response to PEEP of patients with COVID-19 with those of matched ARDS from other etiologies, who have undergone the same procedures.

In our study, respiratory mechanics was highly heterogenous both in COVID-19 patients and in ARDS of other etiologies. Average values of respiratory system compliance were slightly higher in COVID-19 patients. Albeit statistically significant, mean difference (7 ml/cmH_2_O) may not be clinically relevant. This may depend on the small differences in body mass index between the two cohorts, and heterogeneous ARDS causes in the control group. In COVID-19 patients, compliance reduction was linearly related to oxygenation impairment: this indicates that aeration loss is a causative mechanism of hypoxemia, which is the hallmark of ARDS pathophysiology (i.e., the baby lung) [35–38].

Gattinoni et al. have hypothesized that the acute respiratory failure caused by COVID-19 is a time-related disease spectrum within different phenotypes [18]. Our results indicate that, soon after intubation, heterogeneity and average values of respiratory mechanics are similar to ARDS of other etiologies. Our data come from a limited sample. However, results appear consistent with those of a recent large study on 742 patients [39] and with the recently published physiologic data by Haudebourg and coworkers [19]. They found no major differences in respiratory mechanics between patients with ARDS from COVID-19 and other etiologies. They did not exactly match their patients as done in our study, but could not detect relevant differences between ARDS from COVID-19 and other etiologies. Also, other authors have reported high heterogeneity in the respiratory mechanics and response to PEEP of COVID-19 patients [22, 40, 41]. These considerations strengthen the hypothesis that, from a ventilatory standpoint, clinicians should approach COVID-19 patients who fulfill ARDS criteria with our current evidence-based practices, informed by bedside physiology [15, 42–44].

Whether the microvascular involvement represents a disease-specific feature of COVID-19 disease is debated [27, 45]. In our study, ventilatory ratio was slightly higher in COVID-19 patients than in ARDS of other causes. The ventilatory ratio is correlated with dead space and can reflect microvascular thrombosis, which yields ventilation-perfusion mismatch [22]. However, microcirculatory involvement and increased dead space are hallmarks of ARDS as well [46, 47]. Larger cohorts will be needed to subtle differences on this specific aspect.

### Response to PEEP—gas exchange

More than 95% of patients improved oxygenation with high PEEP, independently from recruitability. The oxygenation improvement achieved with high PEEP was greater in COVID-19 patients than in patients affected by ARDS of other causes, although the potential for lung recruitment was not different. PEEP-induced improvement in oxygenation without alveolar recruitment could be caused by decreased cardiac output, with redistribution of lung perfusion towards the normally aerated compartment [22, 34]. This indicates that, similarly to ARDS from other causes, the oxygenation response to PEEP is not informative about alveolar recruitment in COVID-19 as well.

Interestingly, PEEP-induced alveolar recruitment was correlated with PaCO_2_ changes, and all patients with low potential for lung recruitment developed increases in PaCO_2_ with high PEEP. Changes in PaCO_2_ due to PEEP reflect dead space modifications. In case of poorly recruitable lungs, alveolar dead space increases due to compression of pulmonary vessels [48], and airway dead space augments due to gas compression in the respiratory circuit and airways [32]. With alveolar recruitment, overdistension by tidal volume is mitigated, and this reduces ventilation-perfusion mismatch [49, 50].

### Response to PEEP—recruitability

In our study, recruitability of COVID-19 patients was variable, with an average value similar to ARDS from other etiologies. Our results are consistent with most recent data indicating great heterogeneity in the response to PEEP in COVID-19 patients [19, 22, 23]. This has relevant clinical implications, as PEEP setting should balance between its capability to recruit new alveoli and the unavoidable overdistension in already open tissue [51, 52]. As such, a high PEEP should be beneficial only in patients having greater potential for lung recruitment, in whom PEEP increases the size of the aerated lung available for tidal ventilation. Conversely, in non-recruitable patients, PEEP only enhances lung injury by increased static stress and strain [50]. Recruitability could not be predicted by changes in oxygenation, compliance, or driving pressure in response to PEEP, which represent popular proposed PEEP-setting strategies [53]. This suggests that bedside assessment of the potential for lung recruitment appears warranted in COVID-19 patients. The recently developed recruitment-to-inflation ratio (which represents recruited volume normalized to aerated lung size) offers a simple, timely, and reproducible assessment of gas recruitment [29]. This may help distinguish patients showing high vs. low recruitability at the bedside, possibly supporting PEEP setting.

### Clinical outcome

In our study, 28-day mortality of COVID-19 patients was higher than that reported for ARDS of other causes in a large multicenter observational study (35% for moderate and 43% for severe ARDS) [14]. We report high mortality rate despite tidal volume, plateau, and driving pressure were within a relatively safe range and prone position was applied in most of the patients. The majority of our patients were intubated after receiving noninvasive respiratory support, which could have selected the most severe population at higher risk for mortality [14, 54–56]. However, the small size of our cohort precludes from further speculation on the reasons for this high mortality.

### Limitations

This study has limitations.

First, our COVID-19 patients were studied within 24 h after endotracheal intubation: it is possible that respiratory physiology varies over time, as suggested by other investigator showing low respiratory system compliance and minimal recruitability at a later stage of COVID-19 ARDS [57]. This reinforces the clinical message of our study, which addresses individualized interventions based on bedside assessment of physiology.

Second, while the matched comparison with non-COVID-19 historical cohort has several strengths, we cannot exclude that uncontrolled individual characteristics of studied patients affected some of study results. In particular, duration of intubation prior to measurements could not be matched, and patients in the control group showed heterogeneous causes of ARDS: both these features may have affected study results.

## Conclusion

Early after establishment of mechanical ventilation, patients with COVID-19 show a conventional ARDS phenotype, with heterogeneity in respiratory mechanics, aeration loss related to the degree of hypoxemia, and inter-individually variable recruitability. Physiological differences between COVID-19 patients and ARDS from other etiologies appear clinically negligible. Until other data emerge, clinicians treating COVID-19 patients should adhere to most recent guidelines regarding ARDS management.

## Supplementary information

**Additional file 1 : Supplementary Table 1**. Individual data of studied patients.

## Data Availability

The datasets used and/or analyzed during the current study are available from the corresponding author on reasonable request.

## Abbreviations

ARDS: Acute respiratory distress syndrome
PEEP: Positive end-expiratory pressure
BMI: Body mass index
ICU: Intensive care unit
