# Supplementary material for: Respiratory physiology of COVID-19-induced respiratory failure compared to ARDS of other etiologies
Source: Crit Care. 2020 Aug 28;24:529. doi: 10.1186/s13054-020-03253-2 (PMC7453378; doi:10.1186/s13054-020-03253-2)
Supplement: Supplementary file 1 — Additional file 1 : Supplementary Table 1. Individual data of studied patients. [file 13054_2020_3253_MOESM1_ESM.docx]

| Supplementary table 1. Individual data of studied patients. | | | | | | | | | | | | |
| --- | --- | --- | --- | --- | --- | --- | --- | --- | --- | --- | --- | --- |
|  | PaO_2_/FiO_2_ (mmHg) | | | Set PEEP (cmH_2_O) | | | FiO_2_ | | | Tidal volume/PBW, mL/kg | | |
|  | COVID-19 | non COVID-19 | Diff. | COVID-19 | non COVID-19 | Diff. | COVID-19 | non  COVID-19 | Diff. | COVID-19 | non COVID-19 | Diff. |
| Match 1 | 54 | 52 | 2 | 6 | 8 | -2 | 1 | 1 | 0 | 6.3 | 6.3 | 0 |
| Match 2 | 59 | 52 | 7 | 5 | 5 | 0 | 1 | 1 | 0 | 6.4 | 6.3 | 0.1 |
| Match 3 | 61 | 63 | -2 | 0 | 5 | -5 | 1 | 1 | 0 | 6.8 | 6.3 | 0.5 |
| Match 4 | 69 | 69 | 0 | 7 | 8 | -1 | 0.9 | 0.8 | 0.1 | 6.4 | 6.2 | 0.2 |
| Match 5 | 80 | 70 | 10 | 5 | 8 | -3 | 0.8 | 0.7 | 0.1 | 6.8 | 6 | 0.8 |
| Match 6 | 91 | 71 | 20 | 5 | 8 | -3 | 0.7 | 0.8 | -0.1 | 6 | 6.9 | -1 |
| Match 7 | 95 | 76 | 19 | 5 | 5 | 0 | 0.7 | 1 | -0.3 | 6.4 | 5.9 | 0.5 |
| Match 8 | 103 | 91 | 12 | 5 | 6 | -1 | 0.6 | 0.8 | -0.2 | 6.3 | 6.7 | -0.3 |
| Match 9 | 105 | 100 | 5 | 5 | 5 | 0 | 0.6 | 0.5 | 0.1 | 6.8 | 6 | 0.8 |
| Match 10 | 106 | 101 | 5 | 5 | 8 | -3 | 0.6 | 1 | -0.4 | 5.7 | 6.4 | -0.8 |
| Match 11 | 108 | 103 | 6 | 5 | 8 | -3 | 0.6 | 0.8 | -0.2 | 5.4 | 6.9 | -1.5 |
| Match 12 | 110 | 103 | 7 | 4 | 7 | -3 | 0.6 | 0.6 | 0 | 6.4 | 6.1 | 0.2 |
| Match 13 | 116 | 107 | 9 | 6 | 5 | 1 | 0.5 | 0.6 | -0.1 | 5.9 | 5.9 | 0 |
| Match 14 | 117 | 112 | 5 | 5 | 5 | 0 | 0.5 | 0.6 | -0.1 | 7 | 6.3 | 0.7 |
| Match 15 | 118 | 116 | 2 | 10 | 5 | 5 | 0.6 | 0.5 | 0.1 | 6 | 7.6 | -1.7 |
| Match 16 | 120 | 117 | 3 | 5 | 5 | 0 | 0.6 | 0.6 | 0 | 6 | 5.9 | 0.1 |
| Match 17 | 122 | 133 | -11 | 5 | 5 | 0 | 0.7 | 0.6 | 0.1 | 6.4 | 5.4 | 1 |
| Match 18 | 125 | 136 | -11 | 0 | 5 | -5 | 0.6 | 0.5 | 0.1 | 6.4 | 5.7 | 0.6 |
| Match 19 | 126 | 138 | -12 | 5 | 8 | -3 | 0.5 | 0.5 | 0 | 7 | 6.2 | 0.8 |
| Match 20 | 130 | 140 | -10 | 5 | 8 | -3 | 0.6 | 0.5 | 0.1 | 6.7 | 5.9 | 0.8 |
| Match 21 | 132 | 144 | -13 | 4 | 5 | -1 | 0.6 | 0.5 | 0.1 | 6.6 | 5.3 | 1.3 |
| Match 22 | 140 | 148 | -8 | 5 | 5 | 0 | 0.5 | 0.5 | 0 | 6 | 5.3 | 0.7 |
| Match 23 | 140 | 152 | -12 | 5 | 5 | 0 | 0.5 | 0.6 | -0.1 | 7.2 | 6 | 1.2 |
| Match 24 | 147 | 160 | -13 | 5 | 5 | 0 | 0.5 | 0.4 | 0.1 | 5.6 | 5.8 | -0.1 |
| Match 25 | 153 | 172 | -19 | 5 | 5 | 0 | 0.5 | 0.6 | -0.1 | 5.6 | 6 | -0.4 |
| Match 26 | 156 | 172 | -16 | 5 | 5 | 0 | 0.5 | 0.6 | -0.1 | 6.8 | 7 | -0.2 |
| Match 27 | 171 | 177 | -6 | 8 | 5 | 3 | 0.5 | 0.6 | -0.1 | 6 | 5.2 | 0.8 |
| Match 28 | 188 | 195 | -7 | 5 | 5 | 0 | 0.5 | 0.6 | -0.1 | 7.3 | 4.8 | 2.5 |
| Match 29 | 190 | 196 | -6 | 5 | 5 | 0 | 0.4 | 0.5 | -0.1 | 5.9 | 5.3 | 0.6 |
| Match 30 | 200 | 200 | 0 | 5 | 5 | 0 | 0.6 | 0.4 | 0.2 | 7.1 | 6 | 1.1 |
| Mean | 121 | 122 | -1 | 5 | 6 | -1* | 0.63 | 0.66 | 0 | 6.4 | 6.1 | 0.3* |
| Individual data are displayed.  *indicates p<0.05 for the comparison between cohorts. | | | | | | | | | | | | |
